# Mode of Action of Dietary Dexamethasone May Not Be Dependent Upon Microbial Mechanisms in Broilers

**DOI:** 10.3390/microorganisms7090346

**Published:** 2019-09-12

**Authors:** Audrey F. Duff, Mikayla F. A. Baxter, B. Danielle Graham, Billy M. Hargis, Lisa R. Bielke

**Affiliations:** 1 Department of Animal Sciences The Ohio State University, Columbus, OH 43210, USA; 2 Department of Poultry Science, University of Arkansas, Fayetteville, AR 72701, USA

**Keywords:** dexamethasone, stress, intestinal health, broilers, enteric bacteria

## Abstract

Dexamethasone (Dex), a synthetic glucocorticoid (GC), in feed has been shown to increase gut permeability via stress-mediated mechanisms, but the exact mode of action on gut barrier function is not fully understood. Stress has been reported to alter the profile and virulence of intestinal flora predisposing for opportunistic disease. This study aimed to evaluate the relationship between dietary Dex and recoverable intestinal microbial profile in broilers to better understand mode of action and refine future uses of the model. Three experiments were conducted that administered Dex-treated feed for one week in conjunction with the antibiotics BMD (bacitracin methylene disalicylate) or Baytril^®^ (enrofloxacin) to evaluate if enteric microbial mechanisms were important in Dex-induced permeability. Serum fluorescein isothiocyanate-dextran (FITC-d) and bacterial translocation (BT) have been reported to increase after Dex treatment and were used to assess gut epithelial leakage. Shifts in bacterial profiles were also measured on selective agar. Combining Dex with BMD or Baytril resulted in increased (*P* < 0.05) serum FITC-d versus Dex-only. Additionally, Baytril did not reduce aerobic BT and bacterial profiles remained similar after Dex. These results suggest a minimal role of intestinal microbes in Dex-induced changes to intestinal barrier function.

## 1. Introduction

The integrity of the gastrointestinal (GI) epithelium is critical in the maintenance of gut health and performance in poultry. The enteric mucosa represents a vital physical and immunologic barrier that facilitates numerous key functions in the gut, such as secretion of mucus, enzymes, and IgA, selective translocation of nutrients, as well as immune tolerance and responsiveness [1,2]. Intestinal permeability is mediated via transcellular and paracellular pathways which are strongly influenced by the distribution and integrity of intraepithelial tight junctions [3,4]. Barrier dysfunction results in nonselective permeability, or “leaky gut”, that exacerbates pro-inflammatory conditions, susceptibility to infection, and inhibition of growth. The intestinal barrier can be compromised by multiple agents, including toxins, pathogens, antigenic molecules, and hormones [2,4,5,6].

Poultry are subjected to numerous stressors over the course of production such as feed changes, handling, feed withdrawal, transport, and temperature extremes, which can cause the release of stress hormones. The intestinal environment is subject to neurohormonal control, and feedback during times of stress that can result in the activation of the hypothalamic-pituitary-adrenal cortical (HPA) axis, which stimulates the production of corticotropin releasing hormone (CRH) and adrenocorticotropic hormone (ACTH) from the hypothalamus and anterior pituitary gland, respectively. Circulating ACTH stimulates the excretion of glucocorticoids (GC), corticosterone in birds, from the adrenal cortex [6,7,8]. Elevated levels of corticosterone in circulation is a diagnostic marker of stress in poultry [9,10], and it is well demonstrated within the literature that chronic periods of high circulating corticosterone can have significant negative impacts on GI integrity and performance in poultry manifested as reduced weight gain, worsened feed conversion ratio, compromised joint and bone health, lower meat quality, and immunosuppression [11,12,13,14,15,16].

Stress can adversely affect gut motility, paracellular permeability, the expression of adhesion molecules, the production of secretory components, and increase susceptibility to infection [17,18,19,20]. Dexamethasone (Dex) is a synthetic GC analogue with known anti-inflammatory and cell-mediated immunosuppressive effects that has been used to mimic stress conditions in animal models of opportunistic diseases, bone pathologies, and nutrient transport [16,19,21,22]. Few studies have examined the direct effects of Dex on enteric permeability in poultry, and their results tend to agree with mouse and human models that have found prolonged GC exposure results in immunosuppression, such as increased heterophil: lymphocyte ratio and decreased secondary lymphoid tissue weight, decreased defenses against luminal bacteria, and barrier dysfunction as measured by increased recovery of marker molecule fluorescein isothiocyanate dextran (FITC-d) in serum [18,22,23,24,25,26]. Shifts in intestinal microbial populations towards a state of decreased diversity and richness, as well as changes in virulence phenotype of enteric pathogens, can also occur in response to stress and have been reported to result in systemic bacterial infection [27,28,29,30,31]. However, little has been reported on the relationship between Dex as a disruptor of intestinal integrity and the potential role of the presiding microbial population. Characterizing a role of resident intestinal microbes in a chronic exogenous GC-mediated stress response could provide insights for therapeutic targets against stress-mediated enteric complications in broilers and better define models under which Dex would be a useful comparative treatment or predisposing factor.

The following set of studies aimed to determine whether the microbial population served a critical function in the physiologic mechanisms by which GC alter intestinal integrity in broilers as measured via recovery values of CFU/g tissue on selective agar. The following three experiments compared broiler chicks fed a standard starter diet, a diet containing Dex, and a diet containing Dex in combination with the antibiotic BMD50 (bacitracin methylene disalicylate; Exp. 1) or Baytril^®^ (enrofloxacin; Exp. 2). Historically, BMD was used as an antibiotic growth promoter in broilers which targets gram-positive bacteria, such as *Clostridia*, *Streptococci*, and *Staphylococci*, and is known to be poorly absorbed by the intestine [32]. Baytril, a broad-spectrum antibiotic which has similarly been used as a therapeutic growth promoter, was selected in order to observe any potential effects of Dex when the intestinal flora was more intensely reduced beyond the scope of BMD alone. Body weight (BW), serum FITC-d leakage, and bacterial translocation (BT) were used as parameters of increased gut permeability. Although FITC-d recovery in Dex fed birds was similar to previous reports, BT results were not replicated. Additionally, antibiotics did not reduce the leakage of FITC-d or prevent the translocation of bacteria to the liver in Dex treated birds. These results suggest that the resident intestinal bacteria were not strongly influenced and had little to no role in the action of Dex on intestinal barrier function when included in the feed for one week at 0.285 ppm or 0.57 ppm.

## 2. Materials and Methods

### 2.1. Serum FITC-d Recovery

Fluorescein isothiocyanate dextran (MW 3-5 KDa; Sigma Aldrich Co., St. Louis, MO, USA) was used as a marker of increased paracellular transport and mucosal barrier dysfunction. Serum levels of FITC-d were detected similarly to Kuttappan et al. [33] and Vicuña et al. [34]. The volume of FITC-d administered was based on bird weight at time of dosing and was delivered via oral gavage. After 2 h (Exp. 1) or 1 h (Exp. 2), birds were euthanized via CO_2_ inhalation and blood was collected from the femoral vein to quantify levels of FITC-d. Blood samples were left to clot at room temperature for approximately 3 h and then centrifuged 2000× *g* for 15 min for serum separation and collection. Serum samples were diluted in phosphate buffered saline (1:4) and fluorescence was measured at 485 nm excitation and 528 nm emission (Synergy HTX, multi-mode microplate reader, BioTek Instruments Inc., Winooski, VT, USA). Fluorescent concentration of samples was retrospectively determined based on a calculated standard curve obtained from known concentrations of FITC-d.

### 2.2. Bacterial Translocation and Recovery

To measure the translocation of enteric bacteria into circulation and shifts in recoverable populations, portions of the liver, ileum (Exp. 3 only), and ceca were collected aseptically in sterile bags, homogenized, and diluted 1:4 wt/vol with sterile 0.9% saline. Ten-fold serial dilutions were made in sterile 96-well plates and samples were plated on tryptic soy agar (TSA; Merck KGaA, EMD Millipore Co. Billerica, MA, USA), MacConkey agar (Becton, Dickinson and Co., Difco, Sparks, MD, USA), and/or CHROMagar Orientation agar (CHROMagar™ Co., Paris, France) for total aerobic translocation, Enterobacteriaceae positive or negative for lactose fermentation, and changes in select bacterial genera, respectively. CHROMagar plates were placed in anaerobic jars with anaerobic packs (AnaeroPack^®^-Anaero, Mitsubishi, Japan) and all plates were incubated at 37 °C for 24 h in order to determine bacterial shifts and recovery reported as Log_10_ CFU/g of tissue.

### 2.3. Experimental Animals

Three in vivo experiments were conducted to determine whether the resident enteric microbial population played a role in the onset of intestinal permeability previously observed following dietary inclusion of dexamethasone. Experiments 1 and 3 were carried out at The Ohio State University Ohio Agricultural Research and Development Center (OARDC) poultry facilities, while experiment 2 was conducted at the University of Arkansas Poultry Health Laboratory. In all the experiments, day-of-hatch (DoH) broiler chicks were obtained from local hatcheries. In experiments 1 and 2, chicks were kept in a single room in separate floor pens with fresh pine shaving litter and in experiment 3, chicks were placed into brooder battery cages with wire flooring. Chicks had ad libitum access to food and water per the Nutrient Requirements of Poultry: Ninth Revised Edition [35]. Ambient temperature and lighting schedules were maintained within age-appropriate ranges throughout all experiments, and all protocols were approved by respective Institutional Animal Care and Use Committees.

#### 2.3.1. Experiment 1: Effect of Dietary BMD50 Supplementation on Dexamethasone-Mediated Changes in Intestinal Permeability

A total of 900 broiler chicks were randomly assigned to non-supplemented control (C_1_), Dex only (DexF_1_; 0.285 ppm), or BMD50 (50 g/lb) plus Dex (BMD+DexF_1_) treatment groups. Each treatment consisted of 12 replicate pens with 25 birds per pen for a total of 300 birds per treatment. All birds were placed on basal starter diets and Dex was supplemented into the feed of respective groups from d7 through d14. Following removal of Dex, birds were switched to standard grower diets. Birds in BMD+DexF_1_ received BMD50 in feed for the duration of the experiment. All birds were weighed at weekly intervals from d7 through d28 at which times 2 birds per pen were orally dosed with FITC-d (4.17 mg/kg). Birds were euthanized 2 h later via CO_2_ inhalation for serum collection and FITC-d recovery.

#### 2.3.2. Experiment 2: Effect of Dietary Baytril Supplementation on Dexamethasone-Mediated Intestinal Permeability and Enteric Bacterial Translocation

A total of 80 chicks were randomly placed into non-supplemented control (C_2_), Dex only (DexF_2_; 0.57 ppm), Baytril (5 mg/150 g), or Baytril plus Dex (Baytril+DexF_2_) treatments with 20 birds per pen and no replicates. All treatments were supplemented in the feed from DoH. The cumulative weight of each pen was recorded prior to FITC-d (4.17 mg/kg) administration to all birds on d7, and birds were euthanized 1 h later via CO_2_ inhalation for serum to measure FITC-d recovery. Liver and ceca were collected from 12 per treatment birds for bacterial enumeration.

#### 2.3.3. Experiment 3: Effect of Dietary Dexamethasone on Intestinal Permeability and Translocation of Culturable, Differential, Enteric Bacteria

A total of 50 chicks were randomly placed into a non-supplemented control (C_3_) or Dex only (DexF_3_; 0.57 ppm) treatment with 25 birds per pen and no replicates. Dexamethasone was administered d5 through d12. All birds were weighed before and after Dex inclusion. On d12, all birds were euthanized via carbon dioxide inhalation for serum FITC-d, liver, ileum, and ceca collection. Intestinal samples were plated on TSA, MacConkey, and CHROMagar Orientation agar to enumerate differential bacteria.

### 2.4. Statistical Analysis

All BW and FITC-d data in experiments 1 and 2 were subjected to Analysis of Variance as a completely randomized design in JMP Pro 12 statistical software (SAS Institute Inc., Cary, NC, 2016), and BT data in experiments 2 and 3 were subject to Chi-squared analysis with significance at χ^2^ > 3.841 to account for recovery that was below detectable levels. Individual birds were considered the experimental unit for the analysis. Serum FITC-d results showed occasional, random values that were not representative of corresponding treatment means. While a precise reason for these data anomalies is not known, it did not appear to be a treatment response similar to reports by Kuttappan et al. [33] and Vicuña et al. [34]. As such, outliers were identified as above or below two standard deviations from the mean based on empirical or 68-95-99.7 rule and trimmed according to Ghosh and Vogt [36]. Results are presented as mean ± standard error, and significant differences among means (Exp. 1 and 2) were determined using Student’s t-test at *P* < 0.05 unless otherwise stated.

## 3. Results

### 3.1. Body Weight

Individual bird weights were measured only in experiments 1 and 3, although experiment 2 did record cumulative pen weight at the conclusion of Dex treatment on d7 (Table 1). Both DexF_1_ and BMD+DexF_1_ birds showed similar average body weight gain (BWG) over the course of Dex, d7–14, at 178.36 ± 1.71 g and 180.87 ± 1.62 g, respectively, which was significantly lower than 295.07 ± 3.48 g in C_1_ group. Following the removal of Dex on d14, BWG for the treated groups in experiment 1 continued to remain significantly lower than C_1_ at all timepoints, consistent with previous accounts of negative impacts of Dex on growth [7,11,22]. Weight gain d14–21 was significantly higher in BMD+DexF_1_ than DexF_1_ birds with observed gains of 351.58 ± 3.62 g and 337.05 ± 3.39 g, respectively. While these results could be attributed a beneficial effect of BMD50, it is unlikely, as the trend was reversed d21–28 with DexF_1_ exhibiting greater (*P* < 0.05) BWG (Table 1).

Experiment 2 recorded total pen weight at the conclusion of the trial on d7 which was used to calculate average BW per bird. Retrospectively calculated BW/bird was numerically highest in the Baytril and C_2_, 155.09 g and 145 g, respectively, and at least 32 g heavier than Baytril+DexF_2_, 112.19 g, and Dex F2, 100.9 g (data not shown), thus demonstrating the effect of DexF_2_ on BWG. The Dex treated groups in experiment 2 only differed from one another by 12 g, again undermining the involvement of intestinal flora. In experiment 3, the BWG of DexF_3_ birds was significantly lower than C3, 117.80 ± 4.18 g and 139.48 ± 8.25 g, respectively, and therefore exhibited similar growth inhibition trends (data not shown). In all the experiments, growth depression occurred in a similar manner whether Dex was included at 0.285 ppm (Exp. 1) or 0.57 ppm (Exp. 2 & 3).

### 3.2. Serum FITC-d Recovery

**Figure 1:**
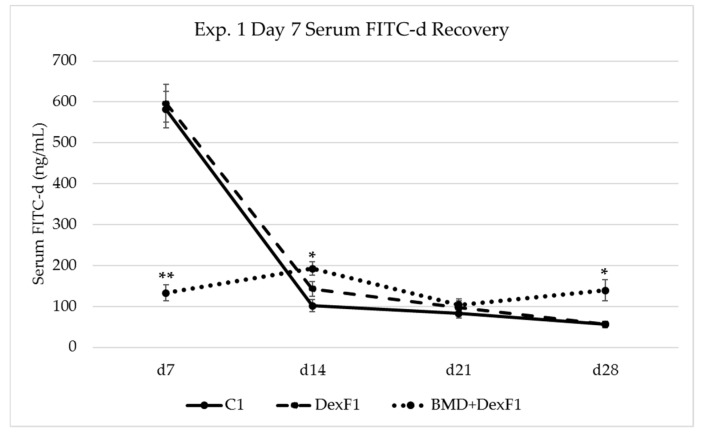
Effect of one week of dexamethasone inclusion in the feed on serum FITC-d recovery from birds in experiment 1. Treated birds received dexamethasone from d7 to d14. Birds were gavaged with FITC-d (4.17 mg/kg) at the indicated time points, and blood samples were collected 2 h later. ** Significantly lower (*P* < 0.001); * Significantly greater (*P* < 0.05). FITC-d sera was diluted 1:4 in PBS onto black 96-well fluorescent plates and measured at 485 nm excitation and 528 nm emission. C_1_ = Control; DexF_1_ = Dexamethasone (0.285 ppm); BMD = Bacitracin methylene disalicylate (50 g/lb); *n* = 24 birds/treatment; Data are expressed as mean ± standard error.

Increased passage of FITC-d into serum was used as an indicator of mucosal barrier dysfunction and increased paracellular transport in experiments 1 and 2. On d7, prior to Dex, C_1_ and DexF_1_ birds exhibited FITC-d recovery levels of 581.01 ± 44.16 ng/mL and 596.48 ± 45.62 ng/mL, respectively, which were higher (*P* < 0.05) than BMD+DexF_1_ at 132.91 ± 20.09 ng/mL (Figure 1). After Dex on d14, BMD+DexF_1_ had significantly greater serum FITC-d recovery, 192.47 ± 16.19 ng/mL, in comparison to C_1_ and DexF_1_ which were 101.54 ± 14.60 ng/mL and 143.06 ± 17.94 ng/mL, respectively. Treated groups had numerically higher FITC-d recovery relative to C_1_ at all timepoints after Dex. While no differences were observed for d21 FITC-d recovery, d28 trends reflected d14 with BMD+DexF_1_ recovery at 138.88 ± 25.92 ng/mL which was higher (*P* < 0.05) than C_1_, 55.65 ± 8.54 ng/mL, and DexF_1_, 56.58 ± 6.39 ng/mL (Figure 1). These variations cannot be explained and are likely due to unknown factors that may have affected mucosal permeability.

**Figure 2:**
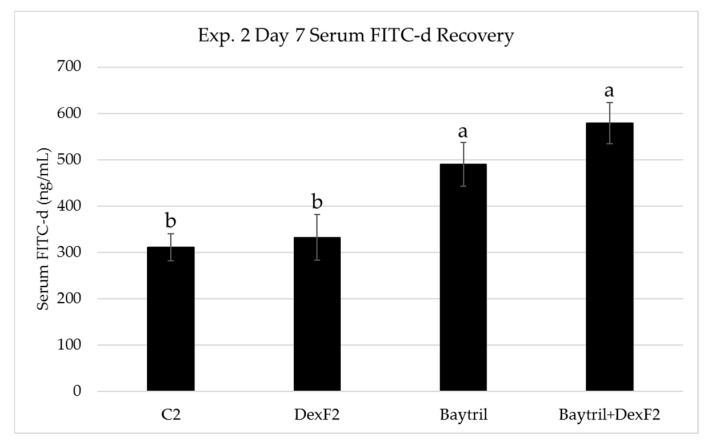
Effect of one week of dexamethasone inclusion in the feed on serum FITC-d recovery from birds in experiment 2. The treated birds received dexamethasone from day-of-hatch to d7. Birds were gavaged with FITC-d (4.17 mg/kg) on d7, and blood samples were collected 1 h later. ^a,b^ Superscripts indicate significant differences at *P* < 0.05. FITC-d sera was diluted 1:4 in PBS onto black 96-well fluorescent plates and measured at 485 nm excitation and 528 nm emission. C_2_ = Control; DexF_2_ = Dexamethasone (0.57 ppm); Baytril inclusion = 5 mg/150 g; *n* = 20 birds/treatment; Data are expressed as mean ± standard error.

Experiment 2 differed from experiment 1 in the magnitude of d7 FITC-d recovery as well observed recovery from antibiotic treated groups although similar trends were noted between C_2_ and DexF_2_. Serum FITC-d was significantly increased from 310.99 ± 22.38 ng/mL and 332.57 ± 49.40 ng/mL in C_2_ and DexF_2_ to 489.99 ± 47.81 ng/mL and 578.88 ± 43.92 ng/mL in Baytril and Baytril+DexF_2_, respectively (Figure 2). However, d7 recovery in experiment 2 followed a similar pattern to d14 recovery in experiment 1, which could indicate differences between the two on d7 were related to timing of Dex treatment.

### 3.3. Bacterial Translocation and Recovery

Livers and ceca were aseptically collected in experiment 2 to measure aerobic BT and shifts in lactose positive and negative populations, respectively. Differences in total aerobic bacteria recovered from the liver on TSA lacked significance but were similar in nature to serum FITC-d recovery, in that recovery from Baytril+DexF_2_ was numerically greater than Baytril which was greater than DexF_2_ (Table 2). Aerobic bacterial recovery from liver of C_2_ birds was higher than expected and dissimilar to FITC-d recovery. Enterobacteriaceae recovery and differentiation of lactose fermenting bacteria on MacConkey agar showed similarly elevated C_2_ values. Significantly more lactose fermenting bacteria were observed in C_2_ and DexF_2_ with respective recovery levels of 7.07 ± 0.26 log_10_ CFU/g and 7.58 ± 0.21 log_10_ CFU/g, in comparison to Baytril and Baytril+DexF_2_ groups with no detected lactose positive colonies. There was also greater (*P* < 0.05) recovery of lactose negative colonies in C_2_, relative to DexF_2_, with values of 7.22 ± 0.19 log_10_ CFU/g versus 5.00 ± 1.10 log_10_ CFU/g, respectively. Both C_2_ and DexF_2_ had significantly more lactose negative colonies than Baytril or Baytril+DexF_2_ which did not exhibit any growth (Table 2).

Aerobic BT was measured in the liver and ceca in Exp. 3 and the recovery levels were numerically similar between treatments (Table 3). A similar lack of differences and similarity between treatment values was noted when examining lactose positive Enterobacteriaceae recovery from the liver, ileum, and ceca. Recovery of lactose negative Enterobacteriaceae in the liver of C_3_ was 0.46 ± 0.21 Log_10_ CFU/g which was greater (*P* < 0.05) than DexF_3_ which had no detectable colonies (Table 3). CHROMagar Orientation agar was used to differentiate bacterial recovery from the liver, ileum, and ceca in order to observe differences in resident populations. After 24 h incubation, the species observed included *E. coli*, *Enterococcus/Klebsiella*, and *Pseudomonas/Staphylococcus*. Birds in C_3_ had significantly higher recovery of *E. coli* in the ileum relative to DexF_3_ with values of 4.56 ± 0.78 Log_10_ CFU/g and 2.29 ± 0.70 Log_10_ CFU/g, respectively (Table 4). Recovery of these species was numerically higher in C_3_ birds for all intestinal sections with the exception of ileal and cecal *Pseudomonas/Staphylococcus* growth.

## 4. Discussion

Activation of the HPA axis and secretion of GC primarily suppresses inflammatory and immune responses through modulation of both the innate and adaptive immune systems [37]. Dexamethasone has been shown to decrease disease resistance and has been used to induce opportunistic diseases in poultry such as colibacillosis, turkey osteomyelitis, and bacterial chondronecrosis with osteomyelitis [16,19,24,38]. Predisposition for these diseases could be related to decreased antimicrobial activity of macrophages observed after administration of GC [39,40]. Additional evidence in the literature suggests that stress-derived shifts in enteric permeability may be associated with signaling between the central nervous system and mucosal mast cells [2,3,6,41]. Activation of the peripheral CRH-mast cell-histamine axis causes degranulation and the release of tryptase, histamine, serotonin and other potentially damaging pro-inflammatory mediators that promote inflammatory conditions, vasodilation and increased vascular permeability [37,42,43]. Future studies determining a more precise role of dexamethasone in broilers could benefit from more thoroughly evaluating the involvement of mast cells as they reside in most all tissues, including the GI tract. Endogenous and exogenous GC can also cause a shift from T helper-1 (Th1) towards Th2 response which favors humoral immunity [37,44]. This shift can lead to susceptibility to infections where cellular immunity is important and has been exploited in GC-mediated models that studied *Eimeria* infection in poultry [38,45]. While Dex has been used successfully as a mediator of opportunistic disease, it is important to consider that many of the previously mentioned studies also inoculated animals with infectious agents to predispose for disease, utilized different routes of delivery and administered various doses.

Despite the methodology in administration of dexamethasone, these studies consistently report decreases in body weight associated with treatment [9,16,31,34]. Similar BWG results were reproduced in all three experiments presented here, indicating that the dietary inclusion models used were adequate for inducing stress associated weight loss. During the week of Dex inclusion in experiment 1, DexF_1_ birds showed nearly identical gain compared to BMD+DexF_1_. These results indicate that the inclusion of BMD neither helped nor inhibited weight gain during chronic exposure to Dex, and suggest that bacteria targeted by the antibiotic were also of little importance in this aspect of the physiologic stress response. This trend was similarly noted in experiment 2. Additionally, BWG during the recovery period, d14–d28, was similar between DexF_1_ and BMD+DexF_1_, which may indicate bacteria targeted by BMD are not an influential component in either weight gain or loss in association with dietary Dex. It is more likely that effects on body weight are the result of altercations in nutrient utilization and changes in fat deposition at the expense of protein, as speculated in the literature [7,11,46,47]. The expected drop in BW and BWG was present in all three experiments, but no particular bacterial species seemed to play a role in this phenomenon after feeding dexamethasone alone or in combination with BMD or Baytril after one week.

Disruptions within the intestinal barrier have been linked to systemic bacterial infections caused by paracellular translocation of enteric pathogens into portal and/or systemic circulation [4,6,26,48]. Fluorescein isothiocyanate dextran is a 3–5 kDa marker molecule that can be used to measure alterations in paracellular permeability between enterocytes. An intact GI barrier does not actively absorb FITC-d, however, during inflammation or other insult to enteric tight junctions, FITC-d can enter circulation. Previous reports have documented increased serum FITC-d recovery in broilers following treatment with dextran sodium sulfate, feed restriction, and Dex [34,49,50]. Our results show that FITC-d recovery was elevated in Dex treated birds, including those administered supplementary antibiotics, relative to controls. (Figure 1 and Figure 2). While numeric trends were similar, the lack of significant differences between DexF and control birds seen in our studies compared with previous works could be attributed to differences in delivery (oral vs feed vs subcutaneous injection), dose, duration, and timing (age of bird) of Dex treatment. Serum FITC-d recovery on d7 in experiment 1 was significantly lower for Dex+BMDF_1_ which was both unexpected and intriguing. One potential hypothesis for this decrease that is currently being further investigated is that BMD supplementation during the first week of life improves gut barrier integrity when the intestine is not fully developed and larger molecules such as maternal antibodies are still actively crossing over [51]. Interestingly, serum FITC-d following Dex inclusion was most elevated in antibiotic fed birds, BMD+DexF_1_, Baytril, and Baytril+DexF_2_. In both experiments 1 and 2, these groups exhibited significant increases in serum FITC-d recovery relative to controls and DexF groups after one week of Dex. Based on these results, feed supplementation with BMD or Baytril did not ameliorate paracellular leakage reported in Dex-fed birds and resulted in increased gut leakage, as measured by FITC-d, relative to Dex alone. While no definite conclusions can be made regarding this antibiotic-related increase in serum FITC-d recovery, similar results have been reported in the literature, particularly when using Baytril [52]. One potential hypothesis for the increased recovery in Baytril+DexF_2_ versus Baytril in experiment 2 is that Baytril, a broad spectrum antibiotic, may have affected the commensal and beneficial microbial populations residing in the intestinal mucosa, and the additional effect of Dex on mucosal physiology and immune cells left the epithelia susceptible to inflammation, infectious agents, or other damage. In poultry, Morales-Barrera and coauthors found that while Baytril had beneficial effects on BWG, serum FITC-d recovery was significantly increased and poults were found to be more susceptible to *Salmonella* Heidelberg infection [52]. There is additional evidence in the literature that antibiotics could negatively impact barrier function in the GI tract [5]. Van Ampting and coauthors noted similar increases in intestinal permeability and *Salmonella* infection following a four-day administration of clindamycin [53]. It is therefore possible that FITC-d results may have been similarly been influenced by BMD or Baytril in experiments 1 and 2 presented here.

Microbial populations have been shown to be susceptible to the influence of stressors in both humans and animals [27,29,30,54]. Growth and virulence promoting interactions have been observed between catecholamines, such as epinephrine and norepinephrine, and bacterial populations such as *E. coli*, *Salmonella*, *Psuedomonas* and *Listeria* [30,55,56]. In experiments 2 and 3 presented here, intestinal sections were plated on MacConkey agar to evaluate changes in recovery of Enterobacteriaceae that were positive or negative for lactose fermentation. Experiment 3, which did not include an antibiotic treatment, showed no differences between C_3_ and DexF_3_ birds in the ceca or ileum, but C_3_ had greater (*P* < 0.05) recovery of lactose negative Enterobacteriaceae in the liver than DexF_3_ (Table 3). Experiment 3 also plated intestinal sections on CHROMagar^®^ in order to evaluate differences in specific bacterial species (*E. coli*, *Enterococcus/Klebsiella*, and *Psuedomonas/Staphylococcus*). Higher (*P* < 0.05) recovery of *E. coli* was observed in the ileum of C_3_ birds which cannot be explained by the authors (Table 4). Experiment 2, which included the broad spectrum antibiotic Baytril, exhibited a significant decrease (no detection) of Enterobacteriaceae in Baytril and Baytril+DexF_3_ relative to C_2_ and DexF_2_ in the ceca (Table 2). It was later speculated that high Enterobacteriaceae recovery in C_2_ may have been due to *E. coli* contamination within the hatcher during a concurrent *in-ovo* experiment. No differences were observed in aerobic BT to the liver in experiments 2 nor 3. While Baytril alone and in combination with Dex resulted in significant decreases in recoverable cecal Enterobacteriaceae, it did not reduce BT to the liver (Table 2). Morales-Barrera et al. showed that enrofloxacin use in poults can result in significant changes to the microbiome and increase the susceptibility to colonization by other groups of bacteria [52]. In this sense, unexpected aerobic translocation to the liver in experiment 2 may be the result of microbiome shifts following Baytril treatment although this is only speculation. However, these results show that increased permeability and BT was not alleviated with antibiotic therapy.

## 5. Conclusions

Results of dexamethasone inclusion in the feed as a mediator of intestinal barrier dysfunction deviated from those previously reported in the literature with regards to BT and to a lesser extent, FITC-d recovery. However, the similar trends in FITC-d and BT results between experiments undermines the idea of a critical role of intestinal microbes in Dex-mediated changes in intestinal permeability. Based on the results obtained from these three experiments, it seems as though Dex may alter intestinal mucosal integrity in a manner that is not dependent on microbial mechanisms but more likely via alterations to the host immune response and mucosal physiology. While enteric bacteria can reportedly exploit a disruption in barrier function and translocate into circulation, the presence of microbes does not appear to be critical in order to observe the effects of exogenously administered GC on body weight and serum FITC-d recovery when dexamethasone is administered in the feed at an inclusion of 0.285 ppm or 0.57 ppm.

## Figures and Tables

**Table 1:** Effect of one week of dexamethasone inclusion in the feed on body weight gain in birds from experiment 1. Treated birds received dexamethasone from d7 to d14.

BWG (g)	d0–7	d7–14	d14–21	d21–28	d14–28
C_1_	135.17 ± 1.76 ^ab^	295.07 ± 3.48 ^a^	446.16 ± 5.92 ^a^	645.24 ± 7.89 ^a^	1089.20 ± 12.71 ^a^
DexF_1_	139.14 ± 1.56 ^a^	178.36 ± 1.71 ^b^	337.05 ± 3.39 ^c^	589.32 ± 5.57 ^b^	926.52 ± 8.37 ^b^
BMD+DexF_1_	133.57 ± 1.37 ^b^	180.87 ± 1.62 ^b^	351.58 ± 3.62 ^b^	564.75 ± 7.24 ^c^	916.26 ± 9.75 ^b^

^a,b^ Superscripts within columns indicate significant differences at *P* < 0.05. C_1_ = Control (Exp. 1); DexF_1_ = Dexamethasone (0.285 ppm; Exp.1); BMD = Bacitracin methylene disalicylate (50 g/lb); *n* = 24 birds/treatment; Data are expressed as mean ± standard error.

**Table 2:** Recoverable bacterial translocation in experiment 2. Recovery of aerobic bacteria from the liver and recovery of lactose positive and negative Enterobacteriaceae from the ceca (limit of detection 500 CFU/g) in 7-day-old broilers after one week of dexamethasone inclusion in the feed. Values are reported as % positive for bacteria (Mean Log_10_ CFU/g ± standard error).

	Bacterial Recovery % Positive (Log_10_ CFU/g of Tissue)		
	Total Aerobic Bacteria (Liver)	Lactose Positive (Ceca)	Lactose Negative (Ceca)
C_2_	33% (0.73 ± 0.32)	92% (7.07 ± 0.26) ^a^	92% (7.22 ± 0.19) ^a^
DexF_2_	8% (0.14 ± 0.14)	83% (7.58 ± 0.21) ^a^	83% (5.00 ± 1.10) ^a^
Baytril	17% (0.33 ± 0.23)	0% (0.00 ± 0.00) ^b^	0% (0.00 ± 0.00) ^b^
Baytril+DexF_2_	25% (0.47 ± 0.25)	0% (0.00 ± 0.00) ^b^	0% (0.00 ± 0.00) ^b^
*P*-value	0.13	<0.001	<0.001

^a,b^ Superscripts within columns indicate significant differences as determined by Chi-squared (χ^2^ > 3.841); C_2_ = Control (Exp. 2); DexF_2_ = Dexamethasone (0.57 ppm; Exp. 2); Baytril inclusion = 5 mg/150g; n = 12 birds/treatment; Aerobic recovery on TSA; Lactose positive/negative Enterobacteriaceae recovery on MacConkey.

**Table 3:** Recoverable bacterial translocation in experiment 3. Recovery of aerobic bacteria from the liver and ceca and recovery of lactose positive and negative Enterobacteriaceae from liver, ileum, and ceca (limit of detection 50 CFU/g) in 12-day-old broilers after one week of dexamethasone inclusion in the feed. For intestinal sections that included samples with no detectable colonies, bacterial recovery values are reported as % positive for bacteria (Mean Log_10_ CFU/g ± standard error).

Bacterial Recovery % Positive (Log_10_ CFU/g of Tissue)								
	Total Aerobic Bacteria		Lactose positive			Lactose negative		
	Liver	Ceca	Liver	Ileum	Ceca	Liver	Ileum	Ceca
C_3_	76% (2.27 ± 0.31)	8.38 ± 0.11	16% (0.80 ± 0.30)	48% (1.54 ± 0.33)	100% (6.26 ± 0.18)	16% (0.46 ± 0.21) ^a^	32% (1.04 ± 0.31)	56% (3.27 ± 0.60)
DexF_3_	68% (2.04 ± 0.30)	8.20 ± 0.26	12% (0.40 ± 0.22)	40% (1.96 ± 0.44)	96% (6.44 ± 0.33)	0% (0.00 ± 0.00) ^b^	13% (0.35 ± 0.19)	48% (3.03 ± 0.63)
*P*-value	0.53	-^†^	0.68	0.57	0.31	0.04	0.09	0.57

^a,b^ Superscripts within columns indicate significant differences as determined by Chi-squared (χ^2^ > 3.841); ^†^ No CFU counts below detectable limits; no Chi-squared *P*-value; C_3_ = Control (Exp. 3); DexF_3_ = Dexamethasone (0.57 ppm); *n* = 25 birds/treatment; Aerobic recovery on TSA; Lactose positive/negative Enterobacteriaceae recovery on MacConkey.

**Table 4:** Recoverable bacterial translocation in experiment 3. Differential bacterial recovery from the liver, ileum, and ceca (limit of detection 50 CFU/g) in 12-day-old broilers after one week of dexamethasone inclusion in the feed. For intestinal sections that included samples with no detectable colonies, bacterial recovery values are reported as % positive for bacteria (Mean Log_10_ CFU/g ± standard error).

Differential Bacterial Recovery % Positive (Log_10_ CFU/g)									
	*E. coli*			*Enterococcus/Klebsiella* ^1^			*Pseudomonas/Staphylococcus* ^1^		
	Liver	Ileum	Ceca	Liver	Ileum	Ceca	Liver	Ileum	Ceca
C_3_	24% (0.87 ± 0.32)	60% (4.56 ± 0.78) ^a^	100% (7.21 ± 0.20)	44% (2.10 ± 0.40)	8.23 ± 0.15	8.90 ± 0.15	8% (0.44 ± 0.25)	0% (0.00 ± 0.00)	28% (1.79 ± 0.60)
DexF_3_	12% (0.65 ± 0.27)	32% (2.29 ± 0.70) ^b^	96% (6.90 ± 0.38)	40% (1.87 ± 0.36)	7.86 ± 0.24	8.80 ± 0.24	8% (0.23 ± 0.16)	4% (0.16 ± 0.16)	20% (1.85 ± 0.61)
*P*-value	0.27	0.05	0.31	0.77	-^†^	-^†^	1.00	0.31	0.51

^1^ Unable to differentiate; ^a,b^ Superscripts within columns indicate significant differences as determined by Chi-squared (χ^2^ > 3.841); ^†^ No CFU counts below detectable limits; no Chi-squared *P*-value; C_3_ = Control (Exp. 3); DexF_3_ = Dexamethasone (0.57 ppm); *n* = 25 birds/treatment; Recovery on CHROMagar Orientation agar.
